# Kinetoplast Genome of *Leishmania* spp. Is under Strong Purifying Selection

**DOI:** 10.3390/tropicalmed8080384

**Published:** 2023-07-27

**Authors:** Evgeny S. Gerasimov, Tatiana S. Novozhilova, Sara L. Zimmer, Vyacheslav Yurchenko

**Affiliations:** 1 Department of Molecular Biology, Lomonosov Moscow State University, 119234 Moscow, Russia; 2 Institute for Information Transmission Problems, Russian Academy of Sciences, 127051 Moscow, Russia; 3 Department of Biomedical Sciences, University of Minnesota Medical School, Duluth Campus, Duluth, MN 55812, USA; 4 Life Science Research Centre, Faculty of Science, University of Ostrava, 710 00 Ostrava, Czech Republic

**Keywords:** genome instability, *leishmania donovani*, *L. infantum*, *L. major*, *L. turanica*, SNP

## Abstract

Instability is an intriguing characteristic of many protist genomes, and trypanosomatids are not an exception in this respect. Some regions of trypanosomatid genomes evolve fast. For instance, the trypanosomatid mitochondrial (kinetoplast) genome consists of fairly conserved maxicircle and minicircle molecules that can, nevertheless, possess high nucleotide substitution rates between closely related strains. Recent experiments have demonstrated that rapid laboratory evolution can result in the non-functionality of multiple genes of kinetoplast genomes due to the accumulation of mutations or loss of critical genomic components. An example of a loss of critical components is the reported loss of entire minicircle classes in *Leishmania tarentolae* during laboratory cultivation, which results in an inability to generate some correctly encoded genes. In the current work, we estimated the evolutionary rates of mitochondrial and nuclear genome regions of multiple natural *Leishmania* spp. We analyzed synonymous and non-synonymous substitutions and, rather unexpectedly, found that the coding regions of kinetoplast maxicircles are among the most variable regions of both genomes. In addition, we demonstrate that synonymous substitutions greatly predominate among maxicircle coding regions and that most maxicircle genes show signs of purifying selection. These results imply that maxicircles in natural *Leishmania* populations remain functional despite their high mutation rate.

## 1. Introduction

Protists of the genus *Leishmania* (Kinetoplastida: Trypanosomatidae: Leishmaniinae) are important causative agents of leishmaniasis, a severe yet neglected human infectious ailment [1,2,3]. The disease has a wide range of clinical presentations, including cutaneous, mucocutaneous, and visceral forms [4,5]. Hundreds of millions of people in over 100 counties are at risk of *Leishmania* infection, with an estimated 1.5 million new cases annually [6].

Overall high levels of genome instability observed in many *Leishmania* species are an intriguing aspect of the parasite’s biology and evolution [7]. Genomic instability is often cited as the key factor that allows *Leishmania* spp. to adapt to a wide range of vectors and vertebrate host species, spreading over dramatically different ecological niches [8,9]. Nuclear genome instability is probably linked to the absence of promoter-dependent gene expression regulation common for all trypanosomatids [10,11]. *Leishmania* DNA instability includes single-nucleotide polymorphisms as well as copy number variation of long genomic regions and even chromosomes (gene dosage) [9,12,13,14,15]. Both have been investigated in the context of identifying mechanisms of virulence and adaptation to hosts [8,16,17].

Because all previously mentioned studies focused on the nuclear genome only, we decided to look at the question of instability, including both the nuclear and mitochondrial (kinetoplast) genomes. Of note, the term kinetoplast refers to a prominent structure containing protein and the DNA of the *Leishmania* single mitochondrion. The kinetoplast genome consists of a few dozen maxicircle molecules (functional analogs of the mitochondrial genome of other eukaryotes) and a few thousand minicircle molecules (encoding small RNAs that guide a unique RNA editing process required for maturation of many maxicircle-encoded genes [18,19].

In the current study, we present an analysis of nuclear and kinetoplast genomes of strains of four closely related *Leishmania* (*Leishmania*) spp. (*L. donovani*, *L. infantum*, *L. major*, and *L. turanica*). Mutations, and specifically, point mutations, are the major playing field of selection and adaptation [20,21]. Using the rate of nuclear and kinetoplast single-nucleotide polymorphisms (SNPs) to estimate the degree of genetic divergence between strains of these four species, we show that their kinetoplast genomes are under strong purifying selection. Relatedly, we characterize the genes located in discrete, fast-evolving regions of *Leishmania* chromosomes.

## 2. Materials and Methods

### 2.1. Datasets

We used raw sequencing data of *Leishmania* strains belonging to four species: *L. turanica* (10 strains sequenced previously by our group [22], BioProject PRJNA888552); *L. major* (12 strains sequenced previously by our group [23], BioProject PRJNA763936); *L. donovani* and *L. infantum* (151 strains [24,25]). Each strain used in the analysis was isolated from a single individual. The accession numbers for sequencing reads used in the current study are summarized in Table S1 for the processed samples that passed quality and coverage controls (described below). All chosen datasets are Illumina paired-end WGS reads with similar read lengths of 100–150 bp.

### 2.2. Data Pre-Processing, Filtering, and Variant Calling

**Figure 1:**
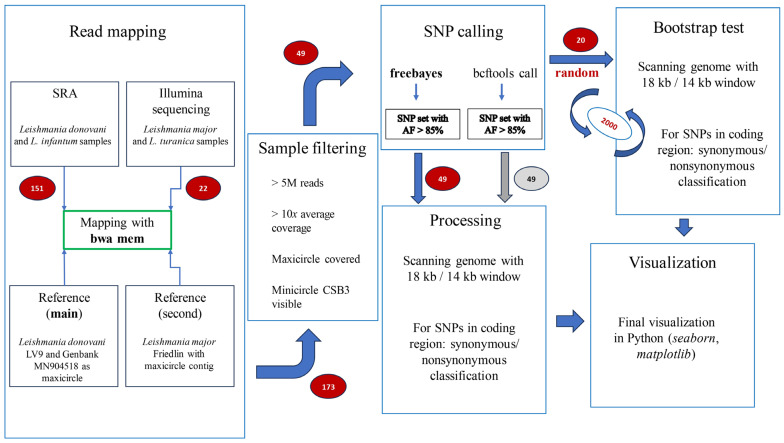
The scheme of the data processing pipeline depicting the main stages. The read mapping was performed for all 173 samples. All analyses were performed independently for both reference genome sequences. *Leishmania donovani* LV9 genome was marked as the ‘MAIN’ reference as all figures in the text are presented for this genome. *Leishmania major* Friedlin was used as the second reference genome. Number of samples (genomes) at each step is provided in red circles. Two SNP callers were used, and the output of both programs was processed identically downstream; blue arrow with number 49 indicates that Freebayes results were used for further presentation in the main text.

Nuclear genomes of *L. donovani* LV9 (TriTrypDB, release 62 [26]) and *L. major* Friedlin (TriTrypDB release 58) were used as references for variant calling. GenBank accession MN904518 and a contig from genome assembly of the strain Friedlin were used as maxicircle references for *L. donovani* and *L. major*, respectively. Sequencing reads were downloaded from SRA with ‘fastq-dump’, and quality control was performed with FastQC v. 6 [27] and MultiQC v. 1.14 [28] tools. Read trimming for adapter sequences and base quality analysis at 3’ ends were performed with Trimmomatic v. 0.32 [29] in the paired-end mode, with minimal retained read length set to 75 bp. Read pairs were mapped onto respective references with Burrows–Wheeler Aligner v. 0.7.17 [30]. Alignment files were processed with SAMtools v. 1.17 [31,32]. Sequencing read libraries were retained for further analysis if they met all the following quality criteria: average maxicircle and nuclear genome coverage over 10×, at least 5 million reads mapped onto the reference genome, at least 25,000 reads containing the CSB3 (Conserved Sequence Block 3) sequence present on minicircles [33], and no coverage gaps of size over 10,000 bp. Those not meeting the criteria were discarded (Figure 1).

Variant calling was performed in BCFtools v. 1.17 [32] and FreeBayes v. 2 [34] with default parameters. Variant calling output files were processed with in-house Python scripts to account for minor formatting differences introduced by SNP callers. Custom Python scripts using scipy, numpy, and seaborn libraries were used for statistical testing and data visualization. Despite different overall numbers of variant calls, both variant calling programs returned very similar results after applying the full data processing pipeline. The distribution of nuclear genome SNPs per 18 kb window is shown in Figure S1 for the Freebayes dataset. The VCF files produced by BCFtools caller were further used to extract homozygous SNPs (AF > 0.85, coverage over 10, variant type = SNP) (Figure 1).

Classification of individual SNPs into categories of those contributing to either synonymous or non-synonymous amino acid substitutions was carried out with an in-house Python script. The program considered the possibility of indels and frameshift mutations, excluding genes with highly modified ORF sequences from further analysis. The script provided accurate per-site information for each annotated gene. Comparison of this script against the published snpEff tool v. 5.1 [35] using both nuclear and kinetoplast datasets showed almost identical results in terms of the total numbers of substitutions, with the snpEff reporting 1–3% more classified substitutions per sample.

### 2.3. Bootstrap Replications

For *L. donovani*, the median number of total synonymous and non-synonymous substitutions was calculated in an 18 kb non-overlapping window for the nuclear genome (average gene content 48.7%) and in and ~18 kb-long coding region (CR) of maxicircles (approximately 52% occupied by non-edited protein-coding genes [18]). Therefore, the size of the scanning window and coding capacity in these datasets is comparable for synonymous/non-synonymous SNP classification comparisons. For *L. major*, the window sizes were 14 kb (the length of its maxicircle CR). To estimate how robust nucleotide substitution median values are, we performed a subsampling (bootstrap), repeating our pipeline on 20 strain libraries randomly taken from a total of 49. We chose the subsample size of 20 to account for possible bias in *Leishmania donovani*/*infantum*- and *L. turanica*/*major*-prevalent subsamples to exclude the possible influence of unequal species representation on the median value.

### 2.4. Functional Annotation and Gene Enrichment

Genomic segment operations (gene and SNP intersection) were performed with BedTools v. 2.31.0 [36]. Genes from variable genome loci were extracted from annotation using custom bash/Python scripts. Gene products were annotated using HMMScan in Pfam v. 35.0 [37] and NCBI-BLAST suite [38]. Gene ontology analysis was carried out with topGO v. 2.46.0 [39] and g:Profiler2 [40].

### 2.5. Selection Analysis

The CODEML program from the PAML package v. 4.8 [41] was used to estimate synonymous and non-synonymous rates (*dS* and *dN*). For the unedited and minimally edited maxicircle-encoded genes (*COI*, *COII*, *COIII*, *ND1*, *ND2*, *ND4*, *ND5*, *MURF2*, and *CYb*), the dN/dS ratio was calculated using M0 model estimation. The guiding tree for analysis was constructed from the full maxicircle CR alignment using FastTree v. 2.1 [42]. To reject possible evidence of positive selection, pairs of nested models M1a-M2a and M7-M8 were also compared with the likelihood-ratio test (LRT).

## 3. Results and Discussion

### 3.1. Whole Nuclear Genome Identification of Point Mutations

The first step in our analysis was to identify point mutations between individual strains of species *L. turanica*, *L. major*, *L. donovani* and *L. infantum,* and a *Leishmania* reference genome. As these four species are closely related [43,44], it would allow us to assess evolutionary changes on a relatively narrow scale of divergence. This will provide opportunities to address questions for which inter-species genetic variability is less suited to compare. These four species are highly syntenic and possess equal numbers of nuclear chromosomes (36). We thus identified strains of these species for which whole-genome sequencing (WGS) reads were available. Libraries for 173 different strains were identified. Out of these, 49 strains met the quality thresholds (See Section 2.2, Table S2: 12 *L. major*, 10 *L. turanica*, 14 *L. donovani*, and 13 *L. infantum*) that would allow SNP analysis of the nuclear and kinetoplast genomes, and the rest were excluded from the analysis. Libraries were mainly discarded due to low maxicircle coverage, indicating that commonly used WGS protocols typically result in low proportions of kinetoplast reads.

Each of the high-quality 49 strains was mapped to the well-annotated reference *L. donovani* LV9 scaffolds for SNP identification. General mapping statistics are presented in Table S2. The number of mapped reads for each strain library ranged from 8 to 56 million reads, with an average nuclear genome coverage between 23 and 225×. We consider this coverage level sufficient for homozygous SNPs calling [45]. An abnormally high percentage of read pairs were mapped onto different chromosomes for three strain libraries, indicative of chromosome translocations in these strains. This is especially pronounced in *L. infantum* IMT373cl1. The total number of homozygous SNPs detected for all 49 strain libraries is reported in Table S2. The percentage of nucleotide mismatch in the study ranged from 0.6 to 1.3% for strains of the more closely related *L. donovani/L. infantum* clade, and from 8 to 9.5% for *L. turanica*/*L. major* strains that are more distantly related to the reference strain to which they were mapped.

### 3.2. Analysis of Kinetoplast Maxicircle Genome Variation

**Figure 2:**
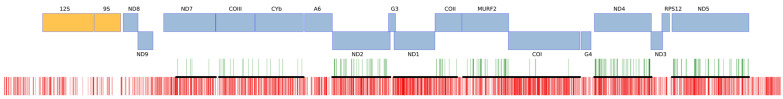
Distribution of SNPs over the *L. donovani* maxicircle CR. (**Top**) Map of *L. donovani* CR. The top and bottom boxes indicate genes encoded on different strands. Color indications for different types of genes: rRNA, orange; unedited genes and cryptogenes, blue. (**Bottom**) The red track shows the positions of all SNPs detected in 49 strains collectively. The green track shows the positions of non-synonymous SNPs (in locations where such a determination was possible). The black segments between tracks indicate maxicircle regions where the determination of synonymous or non-synonymous SNPs can be made. Namely, these are locations where ORFs can be deduced from the maxicircle sequence either because an entire mRNA is properly encoded or else a cryptogene possesses regions that are properly encoded and do not require editing.

The kinetoplast maxicircle of *Leishmania* spp. consists of the variable (divergent) and the coding region (DR and CR, respectively [46]). In the CR, which constitutes nearly half of the maxicircle [~16 of ~36 kb], genetic loci are closely spaced. Uniquely, these loci include traditional genes as well as cryptogenes that generate transcripts that undergo post-transcriptional editing to insert and delete uridines in order to encode their products [11,19]). As an annotated maxicircle sequence was not available to use as a reference scaffold on which to map individual strain reads, for this purpose, we manually annotated the 18 kb CR of *L. donovani* maxicircle (GenBank accession MN904518, sequence length 36,732 bp) with homology-based alignments with maxicircle genes of other *Leishmania* spp. The map is presented in Figure 2.

Strain library reads were subsequently mapped to the newly annotated *L. donovani* CR reference sequence, with an average maxicircle CR coverage ranging from 13 to 318×.

The distribution of SNPs in the maxicircle CR is nonuniform (red track in Figure 2). We then asked whether these SNPs were synonymous or non-synonymous (green track in Figure 2). As it is not possible to assess the eventual functional outcome of SNPs within the edited regions of cryptogenes with DNA information only, these regions were left out of the analysis. A comparison of green and red tracks shows that the vast majority of SNPs in maxicircles are synonymous, approximately 5× more abundant than non-synonymous SNPs. The predominance of synonymous substitutions in maxicircle ORFs indicates that these loci are under strong purifying selection. Estimates of *dN*/*dS* ratios generated with CODEML gave values below 0.03, which implies a strong negative selection pattern. This supports amino acid sequence conservation of maxicircle genes as being vitally important for these *Leishmania* strains collected from the natural environments.

To test for the possibility that positive selection is acting on specific amino acids rather than full gene sequences, we also compared nested site models with CODEML. No selection was detected. Interestingly, *CYb* and *COI* genes are virtually depleted of non-synonymous substitutions, while in *ND2*, *MURF2*, *ND4*, and *ND5* genes, they are enriched relative to the other maxicircle ORF regions. This indicates that the evolutionary pressures on maxicircle gene sequences in *Leishmania* spp. are not uniform for maxicircle genes.

The importance of the various kinetoplast genome-encoded protein products in trypanosomatids is widely analyzed and debated. It is becoming increasingly clear that these parasites retain the capacity to remodel their maxicircles and/or maxicircle mRNA expression in order to adapt to their unique environments. For example, *Phytomonas* and *Vickermania* spp. lack genes encoding *CYb*, *COI*, *COII*, and *COIII* [47,48,49,50,51], apparently having circumvented the need for ATP generation through traditional aerobic respiration. Relatedly, during long laboratory cultivation, *Leishmania tarentolae* can lose groups of minicircles and thus their encoded regulatory RNAs that direct editing, but only those minicircles linked to the editing of specific maxicircle cryptogenes [52]. The maxicircle genes that are no longer able to be edited are likely not essential for *Leishmania* within the context of growth in a nutrient-rich and perturbation-free environment. However, SNP analysis of strains utilized here strongly suggests that the opposite is true in natural isolates of *Leishmania*: their maxicircles display signals of preference for amino acid sequence conservation (low proportion of non-synonymous SNPs), a sign of purifying selection.

### 3.3. Comparison of Nuclear Genome Variation with Maxicircle CR Region Genome Variation

**Figure 3:**
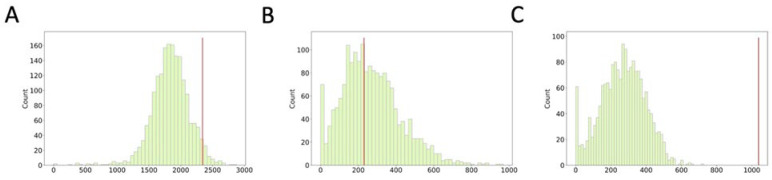
Distribution of nuclear genome SNPs per 18 kb window. Number of SNPs per window is given on the X axis, number of windows with that SNP count is given on Y axis. (**A**) For all SNPs; (**B**) for non-synonymous SNPs (including nonsense); (**C**) for synonymous SNPs. On all histograms, the red vertical line indicates the respective value for the kinetoplast maxicircle CR.

We hypothesized that loci located on nuclear chromosomes have different mutation rates and are under different selection pressures than kinetoplast genome loci. To determine the extent of nuclear genome loci variation in a way that allows us to compare it with those of the maxicircle CR, we investigated synonymous to non-synonymous substitutions of ORFs within 18 kb windows of the nuclear genome, a window length comparable with the length of the maxicircle CR (Figure 3). The ratio of synonymous to non-synonymous substitutions in the nuclear genome is close to 1 (both values have similar distributions with means of 268 and 269 and median of 271 and 251, respectively). This sharply contrasts with the predominance of synonymous substitutions we found for maxicircle CR region loci (1038 synonymous versus 230 non-synonymous ones). Interestingly, the number of non-synonymous substitutions in maxicircles is located near the median value of that of 18 kb windows of the nuclear genome. The rate of amino acid substitutions due to SNPs in both genomes is actually similar. However, the DNA sequence of maxicircles is more likely to accumulate nucleotide substitutions over time (Figure 3A), as most maxicircle CR point mutations are synonymous (Figure 3C). We speculate that the kinetoplast DNA of *Leishmania* may be more prone to damage than its nuclear DNA due to mitochondrial processes that generate reactive oxygen species. Additionally, repair machinery that acts on the nuclear genome may ensure that fewer replication errors become nucleotide substitutions in later generations. In these circumstances in which maxicircle CR SNPs occur at a high rate, purifying selection acting on kinetoplast genes is the cleanest explanation for the modest rate of SNPs resulting in non-synonymous substitutions.

Our conclusion of strong purifying selective pressure on maxicircle coding loci in *Leishmania* is apparently not a phenomenon that applies to kinetoplast genomes in general. Analysis of the monoxenous trypanosomatids *Crithidia bombi* and *C. expoeki* parasitizing social bumblebees has shown that nucleotides of their kinetoplast genomes acquire fewer substitutions than their nuclear genomes [53]. In that work, SNP frequency was measured as the average number of nucleotides in which one SNP is encountered. If we apply the same approach to the current dataset, the averaged values for the *L. donovani*/*infantum* species complex will be 207 and 102 bp *per* SNP for nuclear and kinetoplast genomes, respectively. Thus, even when utilizing this alternative approach, we still conclude that the *Leishmania* strains analyzed acquire more nucleotide substitutions in their maxicircle CR than in their nuclear genome. We conclude that relative evolution rates for kinetoplast and nuclear genomes differ among members of Trypanosomatidae.

Considering that 80% of SNPs in *Leishmania* maxicircles are synonymous substitutions, it is interesting that a recent paper has reported the capacity for synonymous substitutions to be adaptive [54]. They can impact RNA folding and stability, modulate transcription and translation rates, or affect codon usage, which can influence the translation rate. The latter is especially important for trypanosomatid maxicircles lacking identifiable transcription regulatory elements.

### 3.4. Robustness of the Results

The results presented here are not likely to be the result of reference genome bias. When the genome assembly of *L. major* Friedlin rather than that of *L. donovani* was used as a reference, similar numbers of SNPs were obtained and nuclear to maxicircle CR SNP ratios were similar (Figure S2 and Table S2). To demonstrate the robustness of the results with regard to strain library selection, we performed 2000 bootstrap replications, randomly choosing 20 samples out of the pool of 49 and repeated SNP identification (using *L. donovani* as reference). For each replication, a median number of SNPs in each 18 kb window for the nuclear genome and the number of SNPs in maxicircle CR was calculated. The distribution of these two values for 2000 bootstrap replicates is shown in Figure S3. Our results demonstrate that independent of the strain libraries utilized for analysis, the median SNP numbers for nuclear genome 18 kb windows are lower than that of the maxicircle CR. As expected, with replicates that utilized only 20 strain libraries, the absolute value of the median number of SNPs is less: by decreasing the sample size from 49 to 20 strain libraries, the median number of SNPs shifted from 1836 to 1733 (~8%).

### 3.5. Analysis of Highly Variable Nuclear Genome Loci

**Figure 4:**
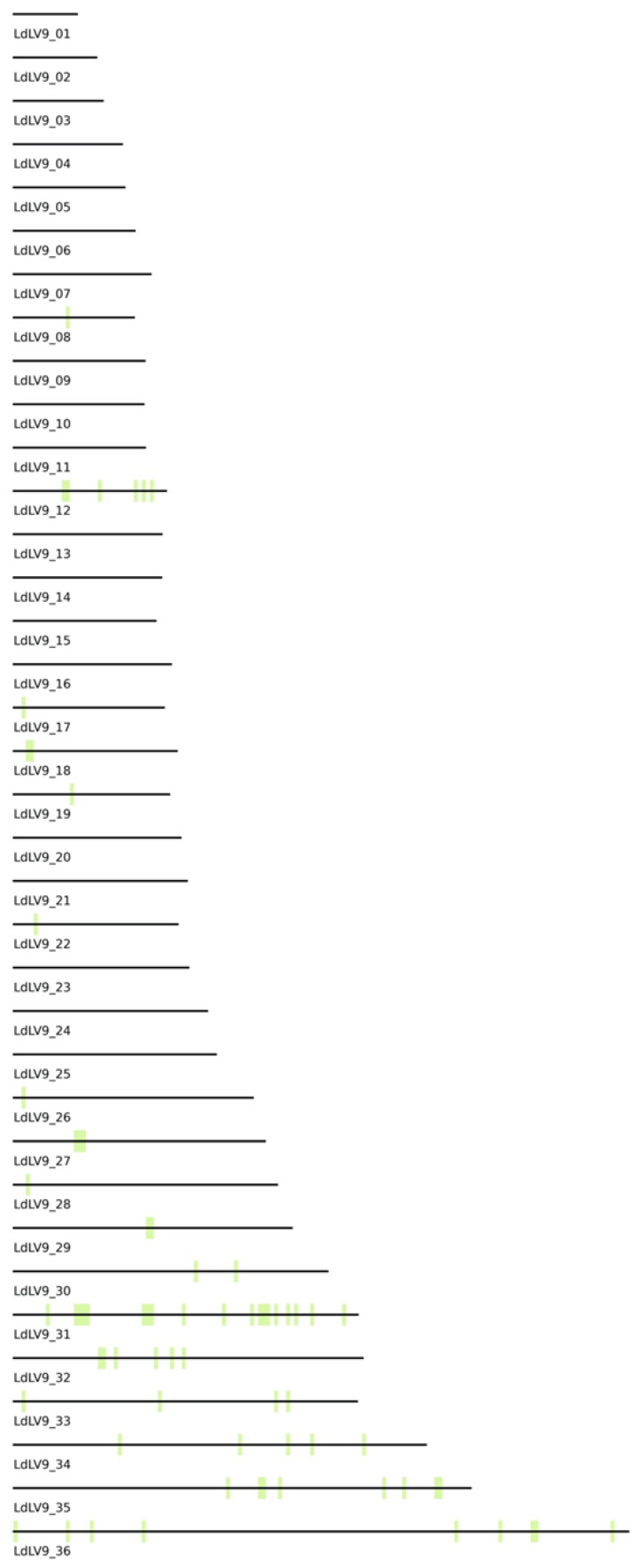
Genomic layout of 18 kb windows with per nucleotide substitution rate equal or higher than that in maxicircle CR. All 36 chromosomes are scaled proportionally to their length.

As can be surmised by Figure 3A, there is variability in the number of SNPs in different nuclear genomic regions. To determine where genomic regions with high nucleotide substitution rates are positioned in the *Leishmania* nuclear genome, we generated chromosome maps on which we plotted the 18 kb genomic windows with total SNP numbers higher than that of maxicircle CRs (Figure 4). These highly variable regions are distributed on the chromosomes in a non-random fashion. Some chromosomes were free of these regions, and others were densely covered. For example, genetically variable regions occupy almost 30% of Chromosome 31. This chromosome is tetra(poly)-somic in most (if not all) *Leishmania* spp. analyzed and is likely involved in recombination processes [55,56,57]. In contrast, we found no direct connection between high SNP-containing windows and chromosome telomeres, centromeres, or repeat-rich regions. The same analysis using the *L. major* genome as a reference resulted in similar distributions with just a few minor differences, most probably caused by fluctuations in window size selection (Figure S4).

Our results show a picture of non-randomly distributed point mutations across the *Leishmania* nuclear genome. Recent findings suggest that genome instability plays an important role in *Leishmania* evolution as it is connected with gene dosage changes that were also shown to be non-randomly distributed across the genome and highly reproducible in populations [7]. Clearly, copy number variations (CNVs) are under selection. Various kinds of repeats and low-complexity regions may act as the drivers for gene dosage through a variety of mechanisms [58]. In *Leishmania* spp. genomes, the association of repeats and CNVs is statistically significant [7]. Conversely, maxicircle CRs lack intergenic regions and, thus, the expression of their genes cannot be regulated by the same mechanism. As discussed above, the synonymous changes we identified have the capacity to modulate translation and, as such, may be adaptive. Based on that, we can hypothesize that the genetically variable regions of the nuclear genome and the maxicircle CR display the same pattern of adaptive evolution.

Finally, we extracted the genes located in the 18 kb windows with high levels of nucleotide polymorphisms and analyzed the likely function of their products to determine if any gene category was enriched among the extracted genes. Homology-based annotations using UniProt and Pfam databases against other annotated trypanosomatid genomes revealed large numbers of genes encoding mitochondria-imported proteins, as well as genes encoding proteins involved in RNA modification and binding, core proteins involved in cell division and mobility, components of the ribosome, and several chaperones. However, formal gene ontology enrichment analysis did not show any significantly enriched categories. Therefore, it is less likely that the identity of the proteins encoded in the regions of the nuclear genome with high nucleotide variability is driving their presence.

## 4. Conclusions

In this work, we investigated nucleotide polymorphisms among 49 *Leishmania* strains and the evolutionary implications of where and in what abundance they were found. The maxicircle CR displays high nucleotide variability, with more SNPs on average than the nuclear genome. This is unexpected and counterintuitive, provided the important functional role the products of maxicircle genes play in trypanosomatid biochemistry. The ratio of maxicircle CR non-synonymous to synonymous substitutions is very low (<0.2), indicating strong purifying selection acting on the *Leishmania* genes encoded in the kinetoplast genome. These results are strong evidence that natural populations of *Leishmania* spp. retain the capacity and a necessity to express functional kinetoplast genes. As laboratory cultures of trypanosomatids can lose their ability to functionally express some kinetoplast genes, these results reveal potential limitations of culture experiments. This is particularly true for the investigation of kinetoplast DNA expression and RNA editing, as these processes initiate from a genome template that may be altered in continuously grown laboratory cultures. Nevertheless, the result obtained for 49 strains of just two species of *Leishmania* must be taken with caution and additional work on other species of Trypanosomatidae (including other species and isolates of *Leishmania*) is warranted in the future. It may also be important to focus attention on the monoxenous trypanosomatids and mutants of the dixenous ones with alterations in their life cycle in order to understand the extent of purifying selection on maxicircles in these species. On a more general note, one way to answer the question of what might affect the high occurrence of synonymous codons is to determine how often these synonymous mutations contribute to adaptation in natural settings beyond the highly contrived laboratory conditions. In other words, does fitness in vitro predict the prevalence of a mutation across a phylogeny? This is a relatively unexplored field, but it was proposed that differential usage of synonymous mRNA variants due to variable speed of decoding on ribosome may be a sign of a “code within the genetic code” for guiding accurate co-translational protein folding and subsequently maintenance of cellular functions [59]. More population-scale sequencing of Trypanosomatidae is needed to address this and related questions [60].

## Data Availability

Publicly available datasets were analyzed in this study. These data can be found here: https://www.ncbi.nlm.nih.gov/ (accessed on 15 June 2023).

## Supplementary Materials

The following supporting information can be downloaded at: https://www.mdpi.com/article/10.3390/tropicalmed8080384/s1, Figure S1: Distribution of SNPs per 18 kb window for nuclear genome of *L. donovani* as reference genome and Freebayes as SNP-caller. The vertical red line denotes the number of SNPs detected by Freebayes in 18 kb maxicircle coding region with flanks. Figure S2: Distribution of SNPs per 14 kb window for the nuclear genome using *L. major* as a reference genome. The vertical red line is the number of SNPs in the *L. major* maxicircle CR; Figure S3: Distribution of the median number of SNPs per 18 kb window for the nuclear genome (using *L. donovani* reference) for 2000 bootstrap replications (sample size 20) is shown in green, and the SNPs median number per maxicircle CR is shown in orange. Green and red vertical lines are medians of these distributions, respectively. Black vertical lines show the median SNP numbers for the nuclear (left line) and maxicircle CR (right line) when all 49 strain libraries are used; Figure S4: Genomic layout of 14 kb windows with *per* nucleotide substitution rate equal or higher than that of maxicircle CR (genomic reference: *L. major*). All 36 chromosomes are scaled proportionally to their length; Table S1: Accession numbers for sequencing reads used in current study; Table S2: General mapping statistics for 49 *Leishmania* strains onto *L. donovani* and *L. major* reference genomes.
