# Supplementary material for: Kinetoplast Genome of Leishmania spp. Is under Strong Purifying Selection
Source: Trop Med Infect Dis. 2023 Jul 27;8(8):384. doi: 10.3390/tropicalmed8080384 (PMC10458658; doi:10.3390/tropicalmed8080384)
Supplement: Supplementary file 1 [file tropicalmed-08-00384-s001.zip › tropicalmed-2503573-suppl-figures.pdf]

Tropical Medicine and Infectious Disease

Supplementary Materials of Kinetoplast Genome of *Leishmania spp.* is Under Strong Purifying Selection

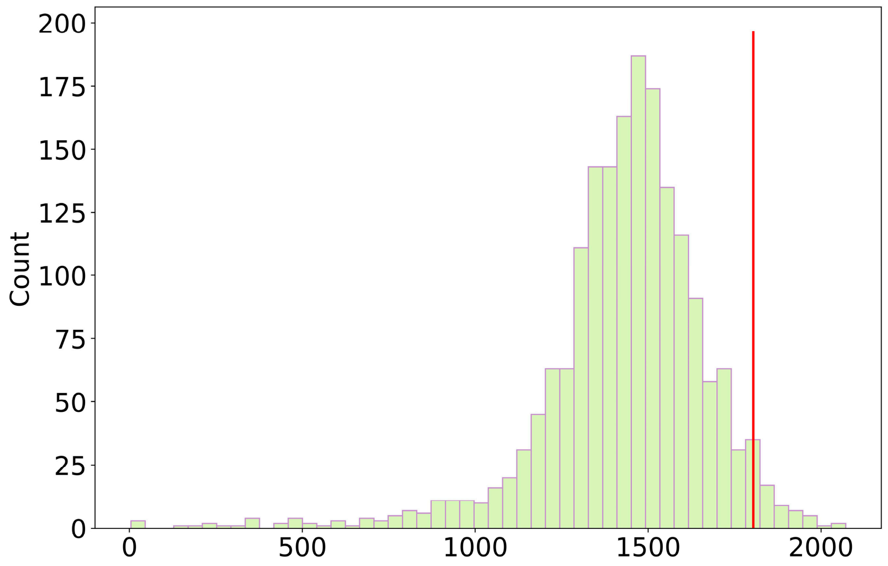

Figure S1. Distribution of SNPs per 18 kb window for nuclear genome of *L. donovani* as reference genome and Freebayes as SNP-caller. The vertical red line denotes the number of SNPs detected by Freebayes in 18 kb maxicircle coding region with flanks.

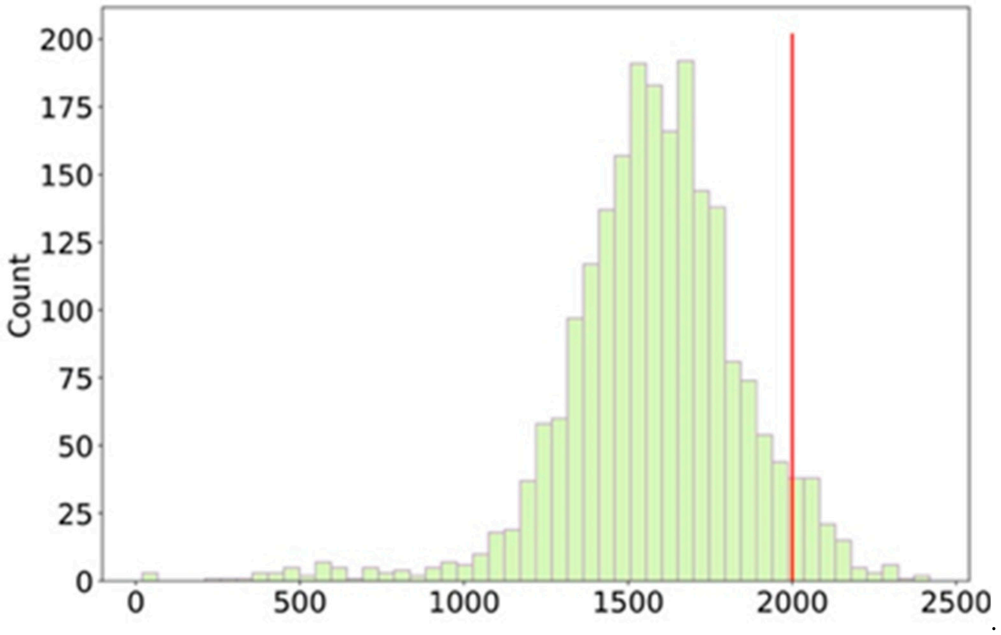

Figure S2. Distribution of SNPs per 14 kb window for the nuclear genome using *L. major* as a reference genome. The vertical red line is the number of SNPs in the *L. major* maxicircle CR.

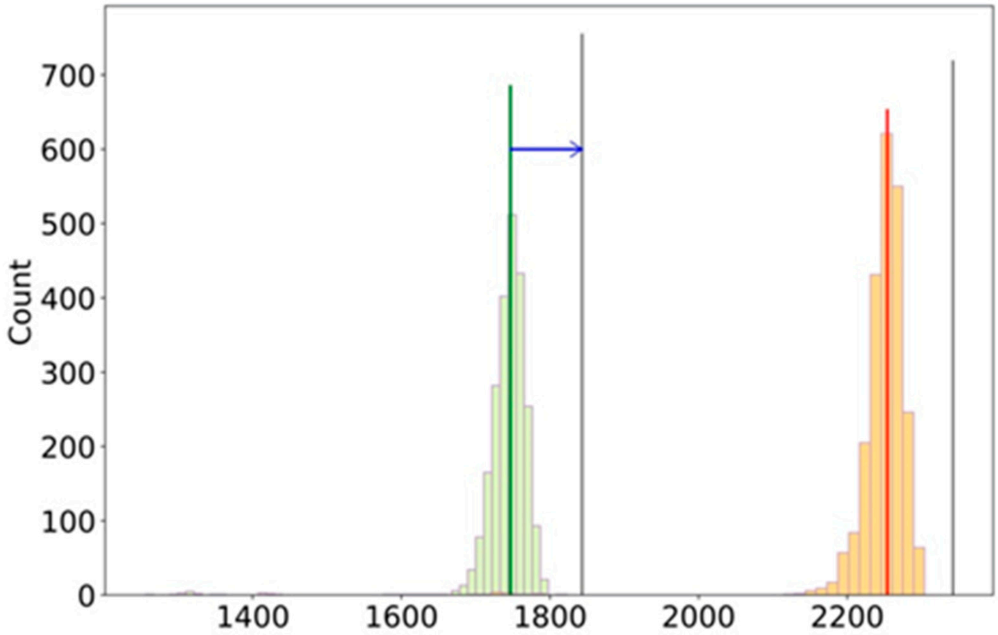

Figure S3. Distribution of the median number of SNPs per 18 kb window for the nuclear genome (using *L. donovani* reference) for 2000 bootstrap replications (sample size 20) is shown in green, and the SNPs median number per maxicircle CR is shown in orange. Green and red vertical lines are medians of these distributions, respectively. Black vertical lines show the median SNP numbers for the nuclear (left line) and maxicircle CR (right line) when all 49 strain libraries are used.

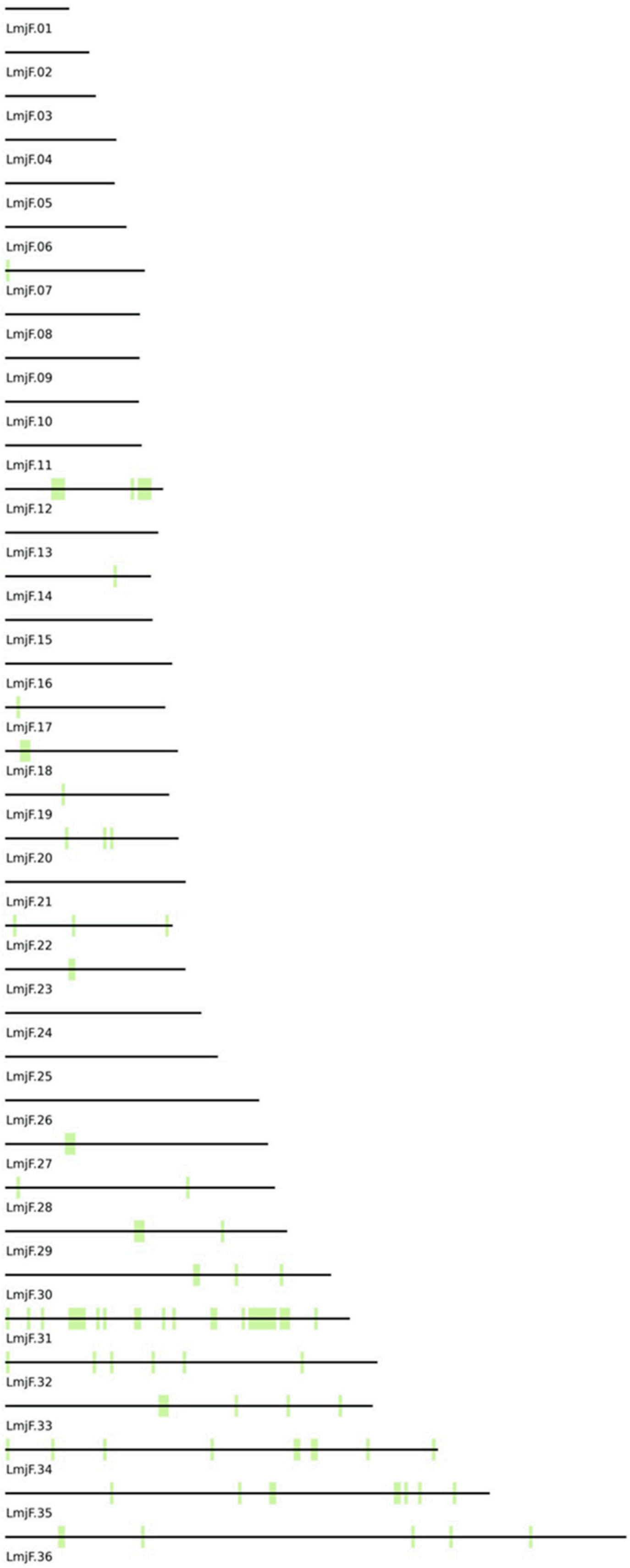

Figure S4. Genomic layout of 14 kb windows with *per* nucleotide substitution rate equal or higher than that of maxicircle CR (genomic reference: *L. major*). All 36 chromosomes are scaled proportionally to their length.
